# Analyzing the gut liver axis: a dual role of the microbiome in the genesis, progression, and treatment of liver cell carcinoma

**DOI:** 10.3389/fmicb.2025.1701101

**Published:** 2025-11-28

**Authors:** Qianzhu Li, Yafang Liu

**Affiliations:** 1 Basic Medical Sciences, Zhejiang Chinese Medical University, Hangzhou, China; 2 Zhejiang Key Laboratory of Blood-Stasis-Toxin Syndrome, Hangzhou, China

**Keywords:** gut microbiota, gut metabolites, gut-liver axis, hepatocellular carcinoma, dual regulation

## Abstract

Hepatocellular carcinoma (HCC) remains a leading cause of cancer-related mortality worldwide, and the poor prognosis highlights the pressing need for innovative therapeutic strategies. The gut-liver axis, a critical bidirectional pathway linking the gut microbiota to the liver, plays a pivotal role in HCC pathogenesis. This review systematically delineates current evidence on how gut dysbiosis, compromised intestinal barrier function, and resultant microbial metabolites (e.g., bacterially metabolized bile acids) drive hepatocarcinogenesis via specific signaling pathways, while also addressing the loss of protective effects due to the depletion of beneficial microbes. Moving beyond descriptive summaries, this article focuses on elucidating the core molecular mechanisms of microbiome-regulated HCC—a key knowledge gap that remains unaddressed—and reconciles conflicting findings into a unified framework. We further explore the translational potential of microbiome signatures as non-invasive biomarkers and evaluate microbiota-targeting interventions (e.g., probiotics, dietary modulation, fecal microbiota transplantation) for enhancing treatment efficacy. Ultimately, this review aims to provide a clear roadmap for developing microbiome-based precision medicine in HCC, with the goal of improving clinical management and patient outcomes.

## 1 Introduction

Hepatocellular carcinoma (HCC) is one of the leading causes of cancer-related mortality worldwide, and its occurrence and progression are closely associated with chronic liver diseases such as viral hepatitis and cirrhosis (Liu Y. et al., 2023). In recent years, the gut microbiota has emerged as a critical regulatory factor in HCC pathogenesis, progression, and therapeutic response through the gut–liver axis, exerting dual effects: it can promote inflammation and carcinogenesis, but may also suppress tumor development via immune-metabolic mechanisms (Seekatz et al., 2018; Huang et al., 2020; Odenwald et al., 2023). However, current research has largely been confined to linear causal relationships between dysbiosis and HCC, lacking systematic integration of microbiome-driven bidirectional immune-metabolic regulation and neuro–immune crosstalk, which limits the advancement of microbiome-based intervention strategies. To address this gap, this review introduces for the first time the concept of a “microbiota–immune–metabolic axis,” emphasizing that microbial metabolites such as short-chain fatty acids and bile acids regulate hepatocyte energy metabolism and the hepatic immune microenvironment, while directly modulating T-cell differentiation and immune checkpoint expression. For example, recent studies have shown that patients with HCV undergoing direct-acting antiviral (DAA) therapy exhibit significant alterations in gut microbiota composition; those achieving sustained virologic response (SVR) display microbial diversity comparable to that of healthy individuals, and genera such as *Elusimicrobium* and Christensenellaceae R-7 are positively correlated with treatment outcomes, suggesting that the microbiota influences therapeutic response through immune modulation (Elsherbiny et al., 2025). Furthermore, this review expands the classical “gut–liver axis” paradigm by incorporating a “microbiota–brain–liver axis” perspective, elucidating how gut microbes interact with the central nervous system via the vagus nerve, immune circulation, and neuroendocrine pathways to regulate hepatic inflammation and the tumor microenvironment. Microbiota-derived metabolites such as GABA and 5-HT have been shown to affect microglial maturation, blood–brain barrier integrity, and hypothalamic–pituitary–adrenal (HPA) axis stress responses, thereby participating in neuro–immune signaling (Ramadan et al., 2025). In summary, this review integrates the dual regulatory mechanisms of the microbiome in HCC through immune-metabolic and neuro–immune pathways, providing new perspectives and theoretical foundations for a deeper understanding of HCC pathophysiology and the development of microbiota-targeted precision therapies.

## 2 Gut microbiota and liver cancer

High-throughput 16S rRNA gene sequencing studies have revealed specific alterations in the abundance of dominant bacterial phyla—Proteobacteria, Actinobacteria, Bacteroidetes, and Firmicutes—within the liver tissue of patients with HCC (Yu and Schwabe, 2017). Among these shifts, the abundance of *Streptococcus maltophilia* is significantly elevated and strongly associated with poor prognosis, whereas the abundance of *Pseudomonas* is markedly reduced. Furthermore, enrichment of *Rhizobiaceae* and *Agrobacterium*, among other taxa, also defines microbial community signatures associated with liver cancer (Dossa et al., 2016). Microbial community composition analysis demonstrates a significant decrease in gut microbial diversity in HCC patients compared to healthy controls. At the genus level, *Parabacteroides*, *Clostridium*, *Gemmiger*, *Phascolarctobacterium*, *Enterococcus*, and other genera show marked increases in abundance (Rajapakse et al., 2023; Behary et al., 2021a; Zhang et al., 2021), changes intricately linked to hepatic inflammatory responses, fibrosis progression, and HCC initiation and development. Conversely, *Verrucomicrobiaceae*, *Bifidobacteriaceae*, *Akkermansia*, *Alistipes*, *Dialister*, and *Collinsella Adlercreutzia* exhibit significant reductions in abundance (Rajapakse et al., 2023; Behary et al., 2021a; Zhang et al., 2021), alterations associated with impaired intestinal barrier function and immune dysregulation, thereby indirectly promoting HCC pathogenesis and progression. Gut microbe-derived trimethylamine N-oxide (TMAO) plays a critical role in the development of various metabolic disorders and malignancies (Zhou et al., 2024). Large-scale case–control studies reveal significantly elevated serum TMAO levels in HCC patients compared to healthy individuals, with increased TMAO concentrations positively correlating with HCC risk (Liu et al., 2018). Gut microbiota dysbiosis exerts a dual influence on liver cancer progression through multiple mechanisms. On the one hand, gut microbiota dysbiosis increases intestinal permeability, promoting the translocation of detrimental bacteria; endotoxins and microbial-associated molecular patterns resulting from bacterial translocation and entering the liver can activate hepatic immune cells, triggering chronic inflammatory responses, and consequently exacerbating liver damage and promoting hepatocarcinogenesis (Yiu et al., 2017). Supporting research indicates positive correlations between circulating flagellin and lipopolysaccharide antibody levels and liver cancer incidence, further substantiating the close association between gut flora imbalance and HCC (Yang et al., 2019). On the other hand, specific gut microbes and their metabolites exhibit inhibitory effects on liver cancer. Probiotics can reduce carcinogen production. SCFAs, metabolites of gut microbes, promote intestinal immune system homeostasis, diminish intestinal inflammatory responses, and suppress the proliferation of pathogenic flora, thereby partially inhibiting HCC initiation and progression (Liu J. et al., 2023). Comprehending the dual mechanisms through which gut microbes and their metabolites influence HCC pathogenesis holds significant clinical implications for HCC prevention, therapy, and personalized intervention. To systematically consolidate these findings, Table 1 provides a comprehensive overview of gut microbiota alterations in HCC patients versus healthy individuals, highlighting community-level dysbiosis, phylum-to-species taxonomic changes, key metabolites (TMAO and SCFAs), immune biomarkers (LPS and flagellin antibodies), and their clinical and pathological significance. This synthesis establishes a foundational framework for the subsequent discussions on the dual mechanisms of microbiome-driven hepatocarcinogenesis.

**Table 1:** Gut microbiota alterations in hepatocellular carcinoma patients compared to healthy individuals.

Category	Microbiota/parameter	Taxonomic level	Change in HCC	Clinical/pathological significance	Refs
Community	Overall gut microbial diversity	Ecosystem	↓Decreased	Dysbiosis, reduced ecosystem stability, and resilience	Rajapakse et al. (2023), Behary et al. (2021a), and Zhang et al. (2021)
Phylum (↑)	Proteobacteria	Phylum	↑ Increased	Liver tissue enrichment; associated with inflammation	Yu and Schwabe (2017)
Phylum (↑)	Actinobacteria	Phylum	↑ Increased	Liver tissue enrichment; pro-inflammatory potential	Yu and Schwabe (2017)
Phylum	Bacteroidetes, Firmicutes	Phylum	Altered*	Metabolic and immune dysregulation	Yu and Schwabe (2017)
Taxa (↑)	Rhizobiaceae	Family	↑ Increased	Microbial signature associated with HCC	Dossa et al. (2016)
Taxa (↑)	*Agrobacterium*	Genus	↑ Increased	Microbial signature associated with HCC	Dossa et al. (2016)
Taxa (↑)	*Parabacteroides*	Genus	↑ Increased	Linked to hepatic inflammation and fibrosis	Rajapakse et al. (2023), Behary et al. (2021a), and Zhang et al. (2021)
Taxa (↑)	*Clostridium*	Genus	↑ Increased	Associated with HCC development and progression	Rajapakse et al. (2023), Behary et al. (2021a), and Zhang et al. (2021)
Taxa (↑)	*Gemmiger*	Genus	↑ Increased	Promotes inflammatory responses	Rajapakse et al. (2023), Behary et al. (2021a), and Zhang et al. (2021)
Taxa (↑)	*Phascolarctobacterium*	Genus	↑ Increased	Contributes to hepatic pathology	Rajapakse et al. (2023), Behary et al. (2021a), and Zhang et al. (2021)
Taxa (↑)	*Enterococcus*	Genus	↑ Increased	Pathogenic potential, inflammation driver	Rajapakse et al. (2023), Behary et al. (2021a), and Zhang et al. (2021)
Taxa (↑)	*Streptococcus maltophilia*	Species	↑ Increased	Strongly associated with poor prognosis	Dossa et al. (2016)
Taxa (↓)	*Pseudomonas*	Genus	↓ Decreased	Loss of potentially protective bacteria	Dossa et al. (2016)
Taxa (↓)	Verrucomicrobiaceae	Family	↓ Decreased	Impaired intestinal barrier function	Rajapakse et al. (2023), Behary et al. (2021a), and Zhang et al. (2021)
Taxa (↓)	Bifidobacteriaceae	Family	↓ Decreased	Reduced protective and anti-inflammatory effects	Rajapakse et al. (2023), Behary et al. (2021a), and Zhang et al. (2021)
Taxa (↓)	*Akkermansia*	Genus	↓ Decreased	Compromised mucus layer integrity	Rajapakse et al. (2023), Behary et al. (2021a), and Zhang et al. (2021)
Taxa (↓)	*Alistipes*	Genus	↓ Decreased	Immune dysregulation	Rajapakse et al. (2023), Behary et al. (2021a), and Zhang et al. (2021)
Taxa (↓)	*Dialister*	Genus	↓ Decreased	Loss of barrier-protective functions	Rajapakse et al. (2023), Behary et al. (2021a), and Zhang et al. (2021)
Taxa (↓)	*Collinsella*	Genus	↓ Decreased	Altered metabolic homeostasis	Rajapakse et al. (2023), Behary et al. (2021a), and Zhang et al. (2021)
Taxa (↓)	Adlercreutzia	Genus	↓ Decreased	Reduced beneficial metabolite production	Rajapakse et al. (2023), Behary et al. (2021a), and Zhang et al. (2021)
Metabolite	TMAO (Trimethylamine N-oxide)	Microbial metabolite	↑ Increased (serum)	Positively correlated with HCC risk; promotes tumor progression	Zhou et al. (2024) and Liu et al. (2018)
Metabolite	SCFAs (Short-chain fatty acids)	Microbial metabolite	↓ Decreased	Loss of anti-inflammatory, barrier-protective, and anti-tumor effects	Liu J. et al. (2023), Guo et al. (2013), and Gao et al. (2021)
Biomarker	LPS antibodies	Immune marker	↑ Increased (serum)	Indicates bacterial translocation and barrier dysfunction	Yang et al. (2019)
Biomarker	Flagellin antibodies	Immune marker	↑ Increased (serum)	Reflects gut-liver axis dysregulation and systemic inflammation	Yang et al. (2019)

Changes indicate alterations in HCC patients compared to healthy individuals. ↑ = increased abundance/levels; ↓ = decreased abundance/levels. *Bacteroidetes and Firmicutes are noted as altered in HCC patient liver tissue, but specific directional changes require further clarification. TMAO = Trimethylamine N-oxide; SCFAs = Short-chain fatty acids; LPS = Lipopolysaccharide. Refs = Reference numbers from manuscript. Color coding: Red = increased taxa; Green = decreased taxa; Yellow = metabolites; Blue = biomarkers.

### 2.1 Mechanisms of gut microbes and metabolites in promoting liver cancer development

**Figure 1:**
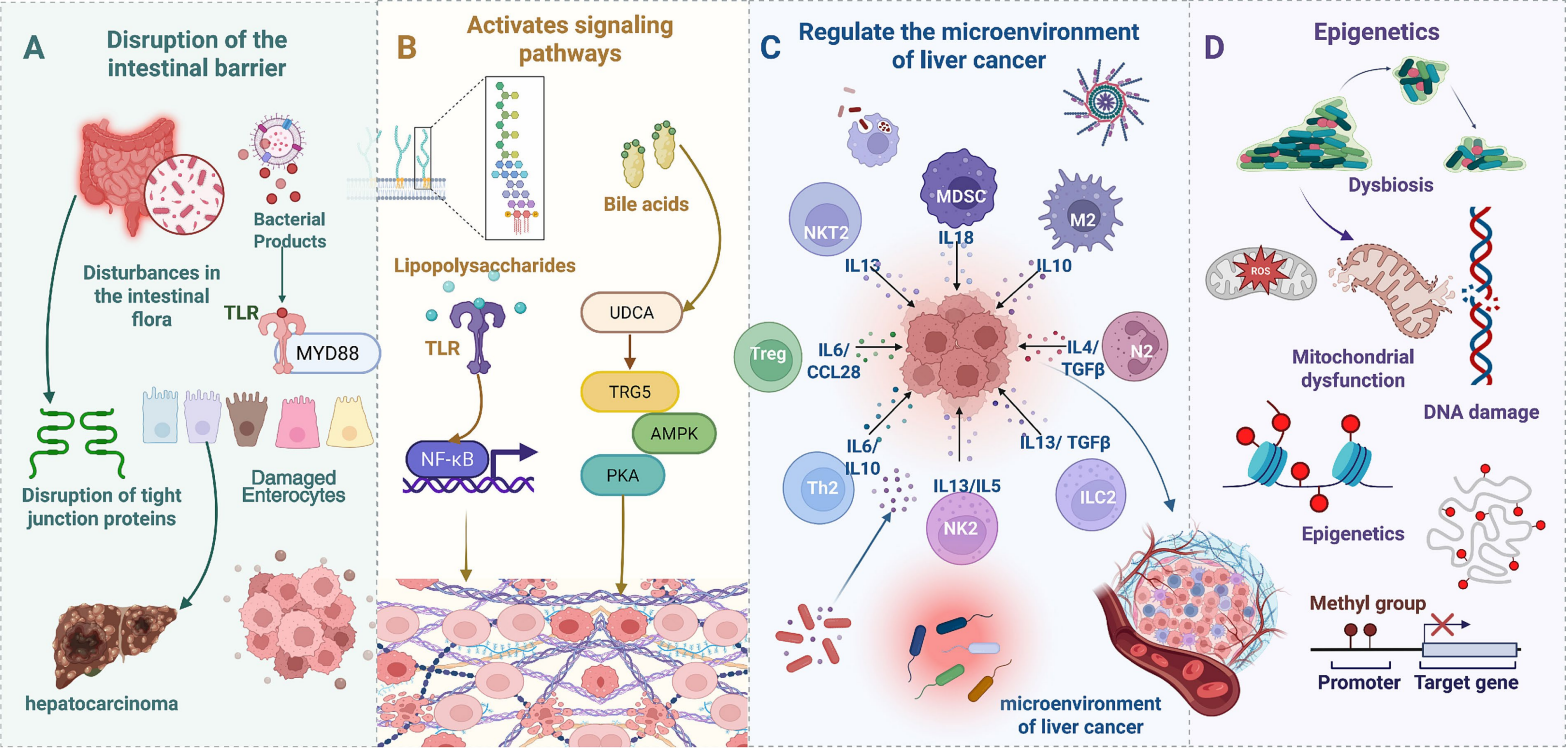
Gut microbes and metabolites in HCC promotion. **(A)** Intestinal barrier disruption. Gut microbiota dysbiosis disrupts the intestinal barrier, compromising tight junction integrity and damaging intestinal epithelial cells, facilitating bacterial translocation into the portal venous system. **(B)** Receptor-mediated signaling. Lipopolysaccharide activates Toll-like receptors on intestinal epithelial cells, triggering myeloid differentiation primary response 88 signaling. Bile acids activate TGR5 receptors, modulating AMPK and PKA signaling pathways. **(C)** Immunemicroenvironment remodeling. Gut microbes and metabolites induce diverse immune cell subtypes (regulatory T cells, T helper 2 cells, myeloid-derived suppressor cells, and natural killer T 2 cells) to secrete immunosuppressive cytokines (IL-13, IL-10, IL-6, TGF-*β*). **(D)** DNA damage and epigenetic modifications. Reactive oxygen species generation leads to mitochondrial dysfunction, DNA damage, and epigenetic aberrations (DNA methylation, histone modifications), promoting liver cancer-associated gene activation or silencing.

Gut microbiota and their metabolites are key regulatory determinants in maintaining liver homeostasis. Dysbiosis of the gut microbiota can induce a range of pathological liver conditions, including liver cancer (Rajapakse et al., 2023). Gut microbial dysbiosis impairs intestinal barrier function and increases permeability, consequently facilitating the translocation of bacteria and ligands into the portal venous system, thereby triggering hepatic inflammatory responses and tissue injury (Ram et al., 2018). In the pathological progression of HCC, dysbiotic gut microbes and their metabolites promote tumorigenesis and advancement through multiple molecular mechanisms, primarily categorized as: induction of DNA damage, regulation of epigenetic modifications, activation of oncogenic signaling pathways, and remodeling of the tumor microenvironment. These elements interact within a complex regulatory network, synergistically fostering HCC initiation and progression, and influencing prognostic outcomes. Figure 1 schematically illustrates the mechanisms by which gut microbes and their metabolites promote HCC development.

#### 2.1.1 Disruption of intestinal barrier integrity

HCC-associated microbial dysbiosis compromises intestinal barrier function, facilitating the translocation of gut-derived bacteria and thereby promoting HCC progression via the gut-liver axis. Gut microbes and metabolites contribute to HCC pathogenesis by disrupting intestinal barrier integrity. Gut microbiota dysbiosis is primarily characterized by an imbalance in the ratio of commensal to pro-inflammatory bacteria and bacterial overgrowth, imbalances that elevate bacterial ligand and endotoxin levels, consequently triggering intestinal inflammatory responses (Rajapakse et al., 2023). Sustained inflammatory responses disrupt epithelial cell tight junctions and brush border structures, impairing intestinal barrier integrity (Maccioni et al., 2020), and leading to heightened intestinal permeability, which enables detrimental microbes and their metabolic products to enter the liver, further inciting hepatic inflammatory responses and promoting hepatocarcinogenesis. Studies have demonstrated a significant association between aberrant elevation of the tight junction protein zonula occludens 1 and increased intestinal permeability, as well as disease progression in HCC patients (Ram et al., 2022). Moreover, endotoxins, such as lipopolysaccharide (LPS), activate the toll-like receptor 4 (TLR4)/myeloid differentiation primary response 88 (MyD88) signaling pathway, inducing myosin light chain kinase upregulation; overexpression of myosin light chain kinase further exacerbates intestinal barrier dysfunction (Nighot et al., 2017; Guo et al., 2021). This cascade intensifies hepatic trafficking of gut microbes and their metabolites, establishing a conducive environment for HCC initiation. *Klebsiella*, a gut pathogen, can translocate to the liver, exacerbating HCC onset and progression. Butyrate, a key gut microbe metabolite, plays a crucial role in maintaining intestinal barrier integrity, suppressing inflammatory responses, and inhibiting tumorigenesis (Guo et al., 2013; Gao et al., 2021). Research indicates that reductions in butyrate-producing flora can lead to impaired intestinal barrier function, thereby promoting HCC progression (Guo et al., 2013). Depletion of butyrate not only weakens the intestinal barrier’s defensive capabilities but also renders the liver more susceptible to the permeation of harmful substances, further fostering a tumor-supportive microenvironment within the liver. To further elucidate this complex interplay, emerging technologies such as the intestine-on-a-chip provide a sophisticated platform to model the gut-liver axis. This system enables the co-culture of microbiota and host cells under physiologically relevant conditions, including oxygen gradients and immune components, thereby facilitating detailed investigation into inflammatory cytokine release and host–microbe interactions (Qi et al., 2023).

#### 2.1.2 Epigenetic modulation

Epigenetic mechanisms, operating through DNA methylation, histone modification, and other pathways, influence gene expression associated with HCC, thereby driving hepatocyte transformation, proliferation, migration, and immune evasion—processes central to HCC pathogenesis (Gao et al., 2024). Gut microbes and their metabolites participate in HCC initiation and progression by modulating epigenetic modifications (Thilakarathna et al., 2021). Bile acids and gut microbes, along with their metabolites, regulate gut microbiota composition through mutual interactions, promote bile acid transformation, and influence the intestinal barrier, immune responses, and epigenetic modifications (Thilakarathna et al., 2021). Primary bile acids, synthesized from liver cholesterol, are conjugated with glycine or taurine before secretion into the duodenum (Chen et al., 2019). Of these, 95% are reabsorbed in the distal ileum into enterohepatic circulation, with the remaining 5% converted into secondary bile acids by *bai*-operon genes under the influence of colonic microbiota (Ma et al., 2018). Secondary bile acids promote the generation of reactive oxygen species (ROS), inducing alterations in DNA methylation patterns and aberrant histone modifications, mediating epigenetic changes, and thereby fostering liver cancer development (Mishra et al., 2024). Prolonged exposure to elevated bile acid concentrations can increase hepatic oxidative stress, which, in turn, can trigger mitochondrial dysfunction, DNA damage, and disruption of cellular membrane structure. These insults further activate signaling pathways such as Ras and nuclear factor kappa-light-chain-enhancer of activated B cells (NF-κB), regulating gene expression via epigenetic modifications, thereby promoting hepatocarcinogenesis (Péan et al., 2013). Mice chronically exposed to a high TMAO environment exhibit a significantly increased incidence of hepatocellular adenoma, a phenomenon closely correlated with TMAO-induced elevations in ROS levels (Shen et al., 2003). Excessive ROS accumulation can induce DNA damage, mitochondrial dysfunction, and the release of inflammatory cytokines, thereby promoting aberrant hepatocyte proliferation and malignant transformation. Furthermore, Gram-positive bacterial lipoteichoic acid, in synergy with the secondary bile acid deoxycholic acid, upregulates cyclooxygenase-2 expression in hepatic stellate cells via activation of the TLR2 signaling pathway and induces the secretion of senescence-associated secretory phenotype factors (Loo et al., 2017). These factors further drive liver cancer progression by inducing the release of pro-inflammatory cytokines, including Interleukin-1β (IL-1β) and IL-6, and modulating oncogene Gro-*α* expression (Yamada et al., 2018).

#### 2.1.3 Activation of relevant signaling pathways

Gut microbes and their metabolites promote HCC formation and progression by activating signaling pathways such as LPS/TLR4 and phosphoinositide 3-kinase/protein kinase B/mechanistic target of rapamycin, thereby influencing hepatic immune responses, inflammatory responses, cell proliferation, and metastasis (Beyoğlu and Idle, 2022). The LPS/TLR4 signaling pathway plays a central regulatory role in this process. Gram-negative bacteria, including *Escherichia coli* and *Klebsiella pneumoniae*, secrete the endotoxin LPS, which binds to TLR4 on intestinal epithelial cell surfaces, initiating the downstream NF-κB signaling pathway. Activation of this pathway enhances the expression of pro-inflammatory cytokines such as IL-6 and tumor necrosis factor-alpha, further inducing chronic inflammatory responses in the liver, promoting hepatocyte proliferation, epithelial-mesenchymal transition (EMT), and tumor metastasis (Dapito et al., 2012). Sustained TLR4 activation not only amplifies hepatic stellate cell activation, leading to increased secretion of growth factors, but also promotes HCC cell survival and immune evasion by altering the liver microenvironment (Dapito et al., 2012). Furthermore, TLR4 activation also triggers broad immune responses and angiogenesis within the liver, a process that further propels sustained HCC development. TMAO can promote HCC cell proliferation, migration, and invasion via the periostin/integrin-linked kinase/protein kinase B/mechanistic target of rapamycin signaling axis (Wu et al., 2022). TMAO can induce overexpression of the matrix protein periostin. Periostin, an extracellular matrix protein, plays a crucial role in promoting tumor cell adhesion, invasion, and drug resistance. Upregulation of periostin can further activate integrin-linked kinase, subsequently promoting HCC cell proliferation, anti-apoptosis, and enhanced invasiveness through the phosphoinositide 3-kinase/protein kinase B/mechanistic target of rapamycin pathway (Wu et al., 2022). The protein kinase B/mechanistic target of rapamycin pathway, a critical pathway in tumor cell growth and metabolic regulation, upon aberrant activation, can enhance cell cycle progression and accelerate HCC progression (Bang et al., 2023).

#### 2.1.4 Modulation of the tumor microenvironment

Upon traversing the portal venous system to the liver, gut microbes and their metabolites can remodel the hepatic tumor microenvironment, thereby potentiating HCC initiation and progression. Liver sinusoidal endothelial cells, critical targets of gut-derived pathogens delivered via the portal vein (Shetty et al., 2018), mediate the localization of Kupffer cells and lymphocytes upon sensing gut microbes, initiating innate immune defense responses (Gola et al., 2021; English et al., 2021). Disruption of this gut-derived immune response consequently triggers hepatic inflammation, inducing the expression of cytokines, chemokines, growth factors, prostaglandins, and pro-angiogenic factors, thereby constructing a pro-tumorigenic microenvironment (Raza et al., 2022). Dysbiosis of the gut microbiota initiates a systemic inflammatory cascade, which subsequently disrupts the functional integrity of intrahepatic immune cells, ultimately accelerating the establishment of an immunosuppressive tumor microenvironment (Behary et al., 2021a; Qi P. et al., 2025). Peripheral blood mononuclear cells from HCC patients undergo transformation toward immunosuppressive T cells, characterized by Treg cell proliferation and functional suppression of CD8 + T cells (Behary et al., 2021b). Furthermore, non-alcoholic steatohepatitis-mediated HCC mouse models demonstrate not only alterations in the intrahepatic immunosuppressive landscape but also an increased number of myeloid-derived suppressor cells (MDSCs) and a concomitant reduction in CD4 + and CD8 + T cell counts, thereby exacerbating liver injury and HCC formation (Schneider et al., 2022). MDSCs, heterogeneous immature myeloid cell populations, are significantly enriched in HCC patients and closely correlate with HCC staging and prognosis. MDSCs promote tumor growth and metastasis while concurrently suppressing anti-tumor immune cell activity. Clinical studies further corroborate that increased Bacteroides abundance is significantly associated with MDSC elevation and increased levels of immune factors, including IL-8 and IL-13. These immune factors orchestrate MDSC recruitment and proliferation, promoting immune evasion and HCC progression (Tobin et al., 2019).

TMAO upregulates the expression of genes such as N-acetylneuraminyl-*β*-galactosidase, layilin, and serine protease high temperature requirement A serine peptidase 3, thereby modulating the HCC tumor microenvironment. N-acetylneuraminyl-β-galactosidase expression is elevated in HCC tissues, potentially promoting cancer cell growth via regulation of tumor cell glucose metabolism and anti-apoptotic signaling (Zhou et al., 2024). Layilin, a transmembrane protein implicated in immune evasion, can potentiate T cell exhaustion and suppress the anti-tumor immune function of CD8 + T cells upon upregulation. High temperature requirement A serine peptidase 3, associated with extracellular matrix remodeling and EMT, accelerates tumor progression by enhancing HCC cell invasion and migration capacity. Furthermore, deoxycholic acid, a gut microbe metabolite, promotes the release of senescence-associated secretory phenotype factors IL-8 and transforming growth factor-β (TGF-β) by inducing senescence of hepatic stellate cells, further exacerbating hepatic inflammatory responses and carcinogenesis (Ram et al., 2022). deoxycholic acid promotes the liver’s pro-inflammatory microenvironment by activating senescence mechanisms in hepatic stellate cells, increasing HCC risk (Rajapakse et al., 2023). Collectively, gut microbes and their metabolites reshape the liver cancer microenvironment, influencing immune cell function and abundance, modulating pro-inflammatory factors and mechanisms of immune evasion, and ultimately driving liver cancer initiation and progression. To provide a systematic overview of these pro-oncogenic mechanisms, Table 2 summarizes the key gut-derived bacteria and metabolites implicated in HCC promotion, along with their molecular mechanisms and supporting evidence levels. This comprehensive categorization highlights the multifaceted pathways through which gut dysbiosis contributes to hepatocarcinogenesis, providing a foundation for understanding subsequent protective mechanisms.

**Table 2:** Pro-oncogenic microbes and metabolites in HCC.

Type	Agent	Key mechanisms	Evidence level
Pro-oncogenic bacteria	*Streptococcus maltophilia*	Associated with poor prognosis	Human data
	*Proteobacteria (phylum)*	Increased abundance in HCC	Human data
	*Actinobacteria (phylum)*	Increased abundance in HCC	Human data
	*Parabacteroides, Clostridium, Gemmiger*	Marked increase; linked to inflammation and fibrosis	Human data
	*Phascolarctobacterium, Enterococcus*	Elevated in HCC patients	Human data
	*Klebsiella pneumoniae*	Translocation to liver; promotes HCC	Preclinical
	*Escherichia coli*	LPS production; TLR4 activation	Preclinical
	*Rhizobiaceae, Agrobacterium*	Enrichment defines HCC signatures	Human data
	*Bacteroides (in specific contexts)*	Associated with MDSC elevation	Human data
Harmful metabolites	TMAO	Activates POSTN/AKT/mTOR pathway;promotes proliferation and invasion	Preclinical (human correlation)
	Secondary bile acids (deoxycholic acid)	Induces ROS, DNA damage, hepatic stellate cell senescence; activates TLR2	Preclinical (human correlation)
	Lipopolysaccharide (LPS)	Activates TLR4/MyD88/NF-κB; chronic inflammation	Human & Preclinical
	Lipoteichoic acid	Synergy with bile acids; upregulates COX-2	Preclinical
	Reactive oxygen species (ROS)	DNA damage, mitochondrial dysfunction, epigenetic alterations	Preclinical

HCC, hepatocellular carcinoma; TMAO, trimethylamine N-oxide; LPS, lipopolysaccharide; TLR, Toll-like receptor; ROS, reactive oxygen species; MDSC, myeloid-derived suppressor cells; POSTN, periostin; AKT, protein kinase B; mTOR, mechanistic target of rapamycin; COX-2, cyclooxygenase-2; NF-κB, nuclear factor kappa-light-chain-enhancer of activated B cells. Evidence Level: Human data—findings from clinical studies or epidemiological research in human subjects; Preclinical—evidence from in vitro or animal model studies; Human correlation—preclinical mechanisms with supporting human correlational data.

### 2.2 Mechanisms of gut microbes and metabolites inhibiting HCC development

**Figure 2:**
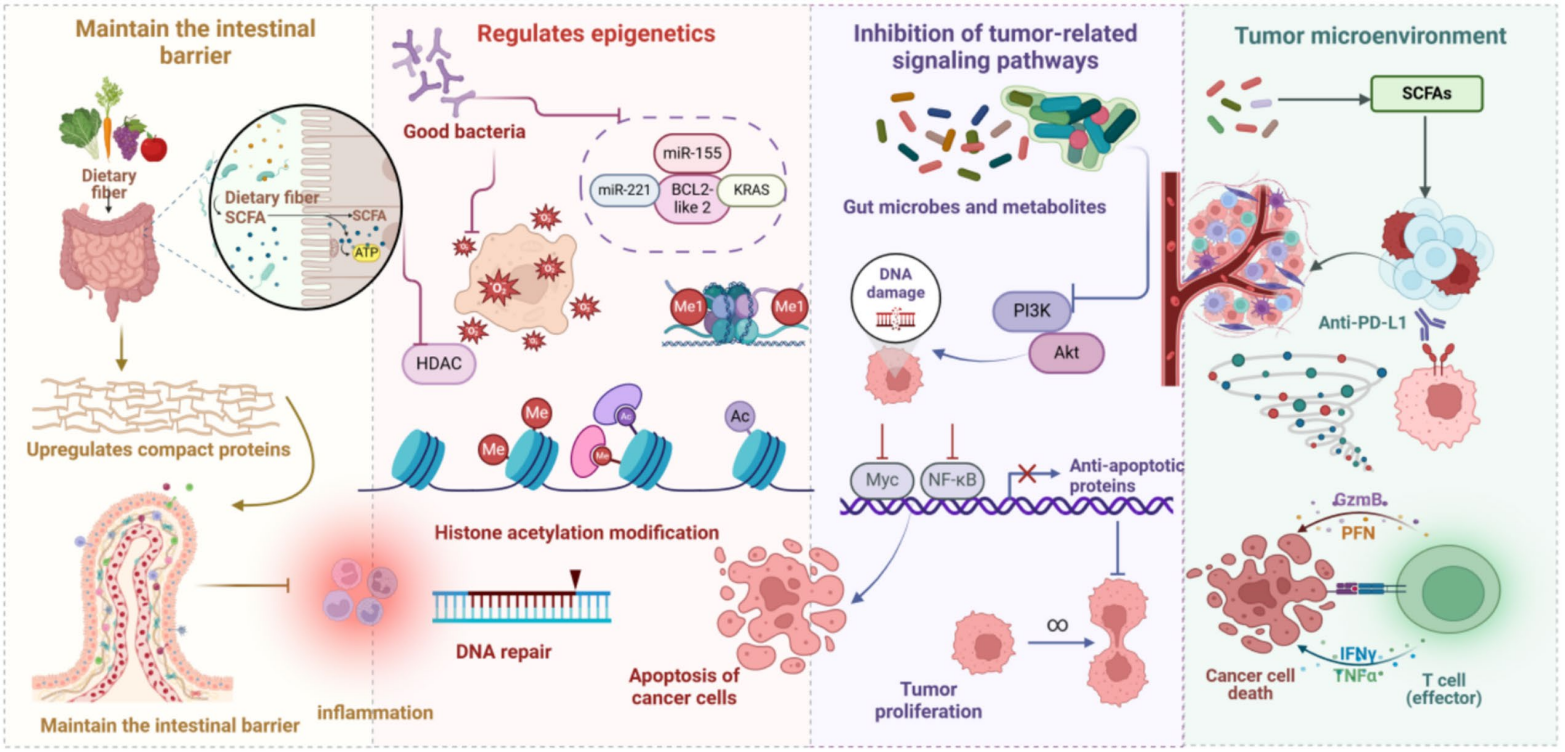
Gut microbes and metabolites in HCC inhibition. Gut microbes, through the fermentation of dietary fiber, yield SCFAs, which fortify intestinal epithelial barrier function by upregulating tight junction protein expression and mitigating intestinal inflammatory responses. This concerted action reduces the infiltration of carcinogenic agents and diminishes the chronic inflammatory milieu within the liver, thereby exerting a suppressive effect on HCC development. Probiotics, by curtailing histone deacetylase activity, augment histone acetylation, thus facilitating DNA repair and promoting cancer cell apoptosis. Moreover, probiotics modulate the expression of key genes and Kirsten rat sarcoma viral oncogene homolog, further influencing cancer cell growth and impeding proliferation. Metabolites derived from gut microbes, by mediating DNA damage repair, modulating the phosphatidylinositol 3-kinase/protein kinase B signaling axis, and suppressing pro-growth signals, such as MYC proto-oncogene, bHLH transcription factor and NF-κB, consequently restrain HCC cell proliferation. Furthermore, gut microbes, by refining the tumor microenvironment and bolstering anti-tumor immune responses, amplify the anti-tumorigenic effects of effector T cells, thereby impeding both the occurrence and progression of HCC.

Beneficial gut bacteria counteract HCC initiation and progression through multiple molecular mechanisms, including maintenance of intestinal barrier homeostasis, regulation of gene epigenetic modifications, suppression of tumor-associated signaling pathway activation, and remodeling of the tumor immune microenvironment. These molecular mechanisms interact, establishing a complex regulatory network that synergistically mediates liver cancer suppression. Figure 2 schematically illustrates the mechanisms by which gut microbes and their metabolites inhibit HCC development.

#### 2.2.1 Maintenance of intestinal barrier function

Probiotics regulate the intestinal microenvironment through multi-layered molecular mechanisms, central to maintaining intestinal barrier integrity (Qi Z. et al., 2025). Their action encompasses both the direct enhancement of epithelial cell junctions and indirect modulation of the immune defense system. On one hand, probiotics and their metabolites upregulate the expression of tight junction proteins such as occludin and claudin, thereby strengthening the epithelial barrier and significantly reducing intestinal permeability. This effectively prevents the translocation of endotoxins, bacteria, and their metabolites into the systemic circulation and their subsequent delivery to the liver, thus alleviating the hepatic metabolic and immune burden (Wan and El-Nezami, 2018). On the other hand, probiotics activate goblet cells to secrete a mucus layer that forms a physical barrier, restricting the adhesion and invasion of pathogens. Simultaneously, they stimulate epithelial immune cells like Paneth cells to secrete antimicrobial peptides, further enhancing the chemical barrier function (Liu et al., 2020). Notably, recent studies indicate that non-nutritive sweetener (NNS) intake may antagonize these protective mechanisms by reducing beneficial bacteria and impairing the production of SCFAs, which are crucial for epithelial energy supply and junction integrity, thereby increasing the risks of intestinal permeability and bacterial translocation (Hetta et al., 2025). Therefore, in microecological interventions for hepatocellular carcinoma, probiotics not only synergistically maintain barrier function and inhibit subsequent hepatic inflammation but also represent a novel approach to correct NNS-induced dysbiosis, thereby blocking the dysregulation of the microbiota-gut-liver axis that drives hepatocarcinogenesis (Wan and El-Nezami, 2018; Hetta et al., 2025).

#### 2.2.2 Epigenetic regulation

Gut microbes and their metabolites exert anti-HCC effects by regulating host gene epigenetic modifications. Gut microbe-host interactions, via epigenetic mechanisms encompassing DNA methylation and histone modification, can modulate host gene transcription levels, thereby profoundly impacting hepatic physiological status and hepatocarcinogenesis (Miro-Blanch and Yanes, 2019). *Lactobacillus acidophilus* and *Bifidobacterium* can suppress HCC cell proliferation and metastasis by downregulating the expression of oncogenes—*miR-155, miR-221, BCL2-like 2,* and *KRAS*. Concurrently, they also promote the expression of tumor suppressor genes—*miR-122* and transcription factor PU.1—thereby maintaining normal hepatocyte function through epigenetic regulation (Thilakarathna et al., 2021). Furthermore, SCFAs by inhibiting histone deacetylase activity, enhance histone acetylation levels, promoting gene transcription and cell differentiation (Lv et al., 2025). This process aids in activating the tumor suppressor gene p53, promoting HCC cell apoptosis, and inhibiting HCC progression. Additionally, SCFAs, via interactions with intestinal macrophages, can suppress the expression of pro-inflammatory cytokines, such as IL-6 and IL-12, and, by increasing histone acetylation levels in the promoter and enhancer regions of the forkhead box protein P3 gene locus, induce regulatory T cell (Treg) differentiation (Fu et al., 2019). forkhead box protein P3, a key transcription factor for regulatory T cells, directly influences immunosuppressive effects and the regulation of inflammatory responses, thereby providing immunological protection against hepatocarcinogenesis (Fu et al., 2019).

#### 2.2.3 Suppression of tumor-associated signaling pathways

Probiotics and their metabolites participate in liver cancer suppression by modulating multiple signal transduction pathways—janus kinase/signal transducer and activator of transcription, TLR/NF-κB, and mitogen-activated protein kinase. The janus kinase/signal transducer and activator of transcription signaling pathway, pivotal in HCC cell proliferation, survival, migration, and immune evasion, activates signal transducer and activator of transcription protein phosphorylation by regulating interactions between cytokine receptors (e.g., IL-6, IL-10, and IL-12) and janus kinase kinases. This cascade subsequently promotes downstream gene transcription, influencing immune responses and tumor progression within the HCC microenvironment (Witalisz-Siepracka et al., 2022). *Bifidobacterium* and *Lactobacillus* genera, by modulating hepatic immune responses, reduce intrahepatic pro-inflammatory cytokine concentrations, including IL-6 and tumor necrosis factor-alpha, attenuating janus kinase/signal transducer and activator of transcription signaling pathway activation, diminishing HCC-associated cytokine expression, and consequently reducing liver cancer cell proliferation and invasion, improving the immune microenvironment, and inhibiting HCC initiation and metastasis. Furthermore, suppression of this pathway elicits NF-κB activity decrease, enhancing cancer cell apoptotic susceptibility (Feitelson et al., 2023). Microbial metabolite-derived polyphenolic compounds can inhibit NF-κB signaling pathway activation, mitigating chronic inflammatory responses and suppressing cancer cell proliferation and metastasis (Williamson and Clifford, 2017). Additionally, probiotics—including *Lactobacillus*, *Lactococcus*, *Streptococcus*, *Enterococcus*, *Bacillus*, *Saccharomyces*, and *Bifidobacterium*—inhibit tumor growth by modulating TGF-*β* (Pourali et al., 2024). TLRs primary host immune system receptors for sensing exogenous pathogens and endogenous damage signals, participate in hepatic immune response regulation. SCFAs by inhibiting TLR4/NF-κB signaling pathway activation, mitigate chronic hepatic inflammatory responses, thereby suppressing hepatocarcinogenesis (Singh et al., 2014). SCFAs reduce TLR4 expression, diminish its responsiveness to endotoxins, and prevent excessive local hepatic inflammatory factor release, consequently reducing cancer cell proliferation, migration, and immune evasion (Singh et al., 2014).

#### 2.2.4 Modulation of the tumor microenvironment

Gut microbes and their metabolites play a significant role in inhibiting HCC initiation and progression by modulating the tumor microenvironment’s immune regulatory network. Experimental investigations substantiate that acetic acid, secreted by *Bacteroides thetaiotaomicron*, enhances cytotoxic CD8 + T cell effector function by modulating pro-inflammatory macrophage polarization, thereby augmenting their anti-tumor activity. Concurrently, fatty acid biosynthesis levels are significantly increased, and acetic acid inhibits pro-inflammatory factor secretion via binding host cell surface G protein-coupled receptors G- protein-coupled receptor 41 and 43 (Sivaprakasam et al., 2016; Luu et al., 2021). Mechanistically, acetic acid inhibits TNF-*α* secretion in monocytes by activating free fatty acid receptors. Acetic acid also promotes M1 macrophage polarization, further enhancing CD8 + T cell cytotoxic effects. Propionate, conversely, inhibits lipopolysaccharide-induced TNF and nitric oxide synthase expression in neutrophils (Masui et al., 2013). Furthermore, SCFAs can also inhibit pro-inflammatory factor production, including IL-6, IL-8, and monocyte chemoattractant protein-1, while enhancing systemic anti-inflammatory effects by inducing IL-10 release, thereby further optimizing the hepatic immune microenvironment (Ohira et al., 2013; Halnes et al., 2017). The novel probiotic compound preparation Prohep exhibits notable anti-tumor effects. This formulation functions by selectively promoting propionate-producing bacteria *Prevotella* and *Oscillibacter* proliferation. *Oscillibacter*, in particular, plays a crucial role in regulating Treg cell IL-10 homeostasis network (Thilakarathna et al., 2021). In tumor-bearing mouse models, Prohep treatment reduced tumor volume by 40%. This anti-tumor effect is primarily mediated by inhibiting Th17 cell population activity and their intestine and peripheral circulation infiltration (Liu et al., 2015). Moreover, Prohep further inhibits HCC growth and metastasis by upregulating anti-inflammatory cytokine expression—IL-10, IL-13, and IL-27 and inhibiting angiogenesis-related factor expression—vascular endothelial growth factor A, fms related receptor tyrosine kinase 1, angiopoietin 2, and kinase insert domain receptor (Li et al., 2016). Probiotics and their metabolites through precise immune cell function modulation, construct an immune microenvironment conducive to HCC suppression. Table 3 provides a comprehensive summary of beneficial gut microbes and their protective metabolites, delineating their anti-tumorigenic mechanisms and the corresponding evidence base. This systematization underscores the therapeutic potential of microbiome modulation and serves as a counterpoint to the pro-oncogenic factors outlined in Table 2, illustrating the dual regulatory role of the gut microbiome in HCC pathogenesis.

**Table 3:** Anti-tumorigenic microbes and metabolites in HCC.

Type	Agent	Key protective mechanisms	Evidence level
Beneficial bacteria	*Bifidobacterium* spp.	Maintains barrier integrity; suppresses oncogenes (miR-155, miR 221, KRAS); reduces inflammatory cytokines	Preclinical (human correlation)
	*Lactobacillus* spp. (*including L. acidophilus, L. rhamnosus* GG*, L. casei*)	Enhances tight junctions; promotes tumor suppressor genes (miR-122, PU.1); modulates JAK/STAT and TLR/NF-κB pathways	Preclinical (human correlation)
	*Akkermansia muciniphila*	Correlated with ICI sensitivity; mucus layer maintenance	Human & Preclinical
	*Verrucomicrobiaceae, Bifidobacteriaceae*	Reduced in HCC; associated with barrier function	Human data
	*Alistipes, Dialister, Collinsella, Adlercreutzia*	Depletion linked to immune dysregulation	Human data
	*Prevotella, Oscillibacter*	Propionate production; regulate Treg/IL-10 homeostasis	Preclinical
	*Bacteroides thetaiotaomicron*	Acetic acid secretion; enhances CD8 + T cell function	Preclinical
Beneficial bacteria	*Lachnoclostridium*	Enrichment increases secondary bile acids beneficial for ICI response	Human data
	*Elusimicrobium,* Christensenellaceae, *R-7Elusimicrobium,* Christensenellaceae *R-7*	Positively correlated with DAA treatment outcomes in HCV patients	Human data
Protective metabolites	Short-chain fatty acids (SCFAs)	Butyrate: Maintains barrier integrity; inhibits HDAC; promotes p53 activation and apoptosis	Human&Preclinical
		Acetate: Enhances CD8 + T cell function; inhibits pro-inflammatory factors via GPR41/43	Preclinical
		Propionate: Inhibits TNF and iNOS; reduces intestinal inflammation	Preclinical
		General: Induces Treg differentiation; increases FOXP3 expression; suppresses TLR4/NF-κB pathway	Human & Preclinical
Protective metabolites	Primary bile acids & FXR agonists	Maintain intestinal barrier; reduce bacterial translocation; inhibit inflammation	Human & Preclinical
	Polyphenolic compounds	Inhibit NF-κB pathway; reduce inflammation and proliferation	Preclinical
	Antimicrobial peptides	Enhanced chemical barrier function	Preclinical

ICI, immune checkpoint inhibitor; SCFA, short-chain fatty acids; HDAC, histone deacetylase; Treg, regulatory T cell; GPR, G protein-coupled receptor; TNF, tumor necrosis factor; iNOS, inducible nitric oxide synthase; DAA, direct-acting antiviral; HCV, hepatitis C virus; FXR, farnesoid X receptor; NF-κB, nuclear factor kappa-light-chain-enhancer of activated B cells; IL, interleukin. Evidence Level: Human data—findings from clinical studies or epidemiological research; Preclinical—evidence from in vitro or animal model studies; Human & Preclinical—convergent evidence from both human and experimental studies. Note: Specific strains within genera may exhibit varying effects; efficacy is context-dependent and influenced by host factors, disease stage, and baseline microbiome composition.

### 2.3 Dual mechanisms and evidence stratification: an integrated analysis of human data, preclinical models, and controversies

In the development and progression of hepatocellular carcinoma (HCC), microbial, metabolic, and immune pathways intersect to produce a suite of dualistic mechanisms that encompass both oncogenic activation and tumor-suppressive regulation; to strengthen mechanistic rigor, we systematically integrated relevant studies and stratified the evidence by source (human data versus preclinical models). On the human data side, large epidemiological studies have demonstrated a significant inverse association between dietary fiber intake and HCC risk—for instance, high consumption of whole grains and dietary fiber is associated with a 22–31% reduction in liver cancer incidence and a 56–63% reduction in chronic liver disease mortality (Liu et al., 2021)—and meta-analyses indicate that each additional 10 g/day of dietary fiber or 16 g/day of whole grains reduces liver cancer risk by 17 and 8%, respectively (Watling et al., 2024); moreover, clinical sample studies support the correlation between elevated serum LPS and flagellin antibodies and HCC risk (Yang et al., 2019), upregulated TLR4 pathway expression in patient liver tissue (Dapito et al., 2012), the construction of diagnostic models based on gut microbiome signatures (Ren et al., 2019), negative correlations between SCFAs and inflammatory markers (Singh et al., 2014), and the ability of FXR agonists to improve liver function and modulate bile acid metabolism (Verbeke et al., 2015; Úbeda et al., 2016), collectively indicating high translational potential that underpins current clinical interventions. By contrast, several mechanisms remain primarily validated in preclinical models, including TMAO activation of the POSTN/AKT/mTOR pathway (Wu et al., 2022), secondary bile acid–induced ROS and cellular senescence (Yamada et al., 2018), probiotic-mediated tumor suppression (Li et al., 2016), and SCFA-driven HDAC inhibition (Woo and Alenghat, 2022); notably, SCFAs exhibit pronounced duality: at low concentrations or under specific metabolic contexts (e.g., acidic tumor pH or hypoxia), SCFAs may promote tumor progression by activating the Hippo–YAP pathway or inducing M2 macrophage polarization (Yang et al., 2024a), whereas at higher concentrations they exert anti-tumor effects—a context dependence confirmed in lung and colorectal cancer models (Ma et al., 2024; Shao et al., 2023) that highlights strong environmental specificity. Preclinical evidence from Yang et al. (2024b) further elucidates this environmental dependence, showing that housing conditions (conventional versus pathogen-free) and drinking water pH directly reshape ketogenic diet-induced gut microbiota (e.g., by enriching Lachnospiraceae and Oscillospiraceae) and metabolic phenotypes in mice, independent of energy intake or ketone bodies. These results highlight how environmental variables modulate microbiome-mediated mechanisms, thereby reinforcing phenotypic dualities analogous to those in HCC pathways (Yang et al., 2024b). Further analysis identifies major mechanistic controversies, particularly regarding bile acids and antibiotics: the former may present pro-carcinogenic effects (secondary bile acids) versus anti-tumor effects (FXR agonists) at different disease stages, while the latter can both improve microecology by suppressing carcinogenic microbiota (Singh et al., 2020) and potentially attenuate the efficacy of immune checkpoint inhibitors (Pinato et al., 2023); SCFAs likewise display dual roles, inducing Tregs and suppressing inflammation yet (Woo and Alenghat, 2022), under specific conditions (such as dysbiosis or fiber-induced metabolic remodeling), potentially promoting tumor progression by increasing oxidative stress or altering bile acid profiles (Feitelson et al., 2023). In sum, the duality of HCC-related mechanisms manifests not only at the functional level but also across evidence sources and model systems: while the protective effect of dietary fiber is established in human epidemiology, specific mechanistic pathways (e.g., the context-dependent roles of SCFAs) remain heavily reliant on experimental models and microenvironmental context, indicating the need for strengthened human validation and explicit delineation of controversies and context dependencies in mechanistic syntheses to enhance clinical relevance and interpretability.

**Figure 3:**
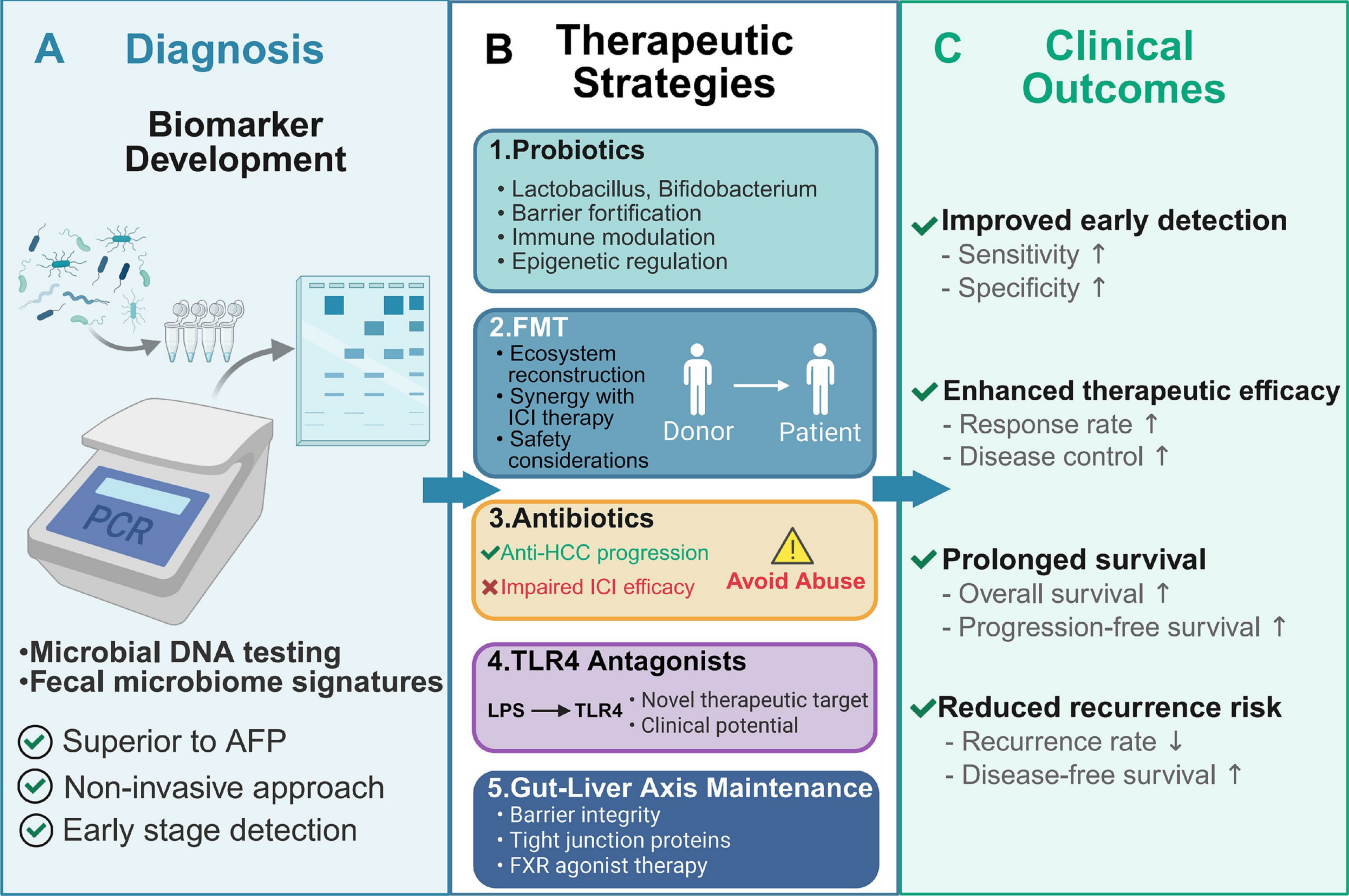
Translational applications of gut microbiome modulation in hepatocellular carcinoma: from diagnosis to therapeutic intervention. This schematic overview illustrates the multifaceted applications of gut microbiome-based strategies in hepatocellular carcinoma (HCC) management, spanning diagnostic innovation, therapeutic modulation, and clinical outcome optimization. **(A)** Fecal microbiome profiling through microbial DNA testing enables non-invasive HCC diagnosis, with characteristic microbial signatures achieving superior diagnostic performance (AUC > 80%) compared to conventional AFP, offering advantages in early detection, sensitivity, and specificity. **(B)** Five therapeutic strategies target the gut-liver axis: (1) Probiotics (*Lactobacillus*, *Bifidobacterium*) restore barrier integrity, modulate immunity, and regulate epigenetics; (2) FMT reconstructs healthy gut ecosystems and synergizes with ICI therapy while requiring careful safety considerations; (3) Antibiotics selectively suppress pro-carcinogenic bacteria but may impair ICI efficacy, necessitating judicious use; (4) TLR4 antagonists interrupt LPS-driven hepatic inflammation as novel therapeutic targets under clinical investigation; (5) Gut-liver axis maintenance strategies preserve barrier function through tight junction proteins and FXR agonist therapy. **(C)** These interventions collectively improve clinical outcomes by enhancing early detection (increased sensitivity and specificity), therapeutic efficacy (elevated response and disease control rates), overall and progression-free survival, and reducing recurrence risk through sustained microbiome homeostasis. This framework represents a paradigm shift toward microbiome-based precision medicine in HCC.

To reconcile these complexities and controversies, a more holistic view that incorporates extra-hepatic organs, particularly the spleen, into the pathogenesis model of HCC is required. When dissecting the systemic immune-metabolic network associated with hepatocellular carcinoma (HCC), it is essential to emphasize the central role of the spleen, the mechanism of which is particularly critical in the cascade of events triggered by intestinal barrier injury. Upon damage to the intestinal mucosa caused by factors such as gut dysbiosis or chemical injury (e.g., DSS induction), microbial products (e.g., LPS) translocate into the systemic circulation. This not only directly drives local hepatic inflammation but also triggers a state of chronic low-grade systemic inflammation. This process elicits a marked response in the spleen—the largest secondary immune organ in the systemic circulation—characterized by splenomegaly, metabolic reprogramming of immune cells (e.g., decreased glycolytic capacity), and increased apoptosis of CD45 + cells, thereby disrupting T-cell homeostasis and exacerbating immune dysfunction (Wang et al., 2023). Such splenic dysfunction further amplifies its pathological impact in the context of non-alcoholic fatty liver disease (NAFLD)—a critical underlying condition for HCC—via the “liver-spleen axis.” Clinical studies have confirmed that an increased splenic longitudinal diameter (SLD) in NAFLD patients is closely associated with insulin resistance and the level of chronic inflammation. The spleen profoundly participates in shaping the hepatic immune microenvironment through mechanisms such as regulating the Th17/Treg balance, B-cell differentiation, and macrophage polarization, thereby influencing the initiation, progression, and therapeutic response of HCC (Tarantino et al., 2021). Consequently, integrating the “liver-spleen axis” perspective into the classical “gut-liver axis” framework provides a more systematic pathophysiological cascade: starting from gut dysbiosis and barrier impairment, through systemic inflammation activating splenic immune-metabolic disturbances, and further via immune crosstalk between the liver and spleen, ultimately driving the malignant progression of HCC (Wang et al., 2023; Tarantino et al., 2021). This offers a novel theoretical basis for understanding the systemic regulatory network of HCC and developing targeted intervention strategies (Figure 3).

## 3 Translational applications of the gut microbiome in liver cancer diagnosis and therapeutics

HCC, a malignancy characterized by poor prognosis, presents persistent therapeutic challenges. Despite surgical resection remaining the primary curative approach, 5-year recurrence rates remain alarmingly high, exceeding 70% (Jiang et al., 2019). Clinical evidence indicates that a majority of patients are diagnosed at advanced stages, precluding radical treatment opportunities (Liu et al., 2015). Current comprehensive treatment modalities, encompassing chemotherapy, radiotherapy, immunotherapy, and targeted therapies, offer limited patient prognosis improvements (Llovet et al., 2022). Therefore, deeper exploration of the molecular mechanisms by which the gut microbiome influences HCC pathogenesis, coupled with assessment of its therapeutic potential, is of paramount scientific and clinical significance for developing novel treatment strategies and improving patient outcomes. This approach is exemplified by recent integrative studies that have identified Lumican (LUM) through convergent bioinformatics and functional validation as a tumor suppressor in HCC, where its overexpression inhibits proliferation, migration, and invasion while inducing apoptosis, underscoring the value of mechanistic discovery (Zhou et al., 2025). Table 4 presents an integrated overview of emerging microbiome-based strategies under investigation, ranging from biomarker development to therapeutic interventions, along with their mechanistic targets, current clinical status, and key considerations for clinical translation. This roadmap guides the subsequent detailed discussion of diagnostic tools (Section 3.1), therapeutic modulation strategies (Section 3.2), and gut-liver axis maintenance approaches (Section 3.3).

**Table 4:** Translational strategies under investigation.

Strategy	Specific approach	Mechanism/target	Clinical status	Key findings/considerations
Biomarker development	30-marker microbial panel	Non-invasive early HCC diagnosis	Clinical validation	AUC 80.64% in distinguishing early HCC from non-HCC
	Serum TMAO levels	Risk assessment	Human correlation study	Positively correlated with HCC risk
	Fecal microbial diversity	Disease progression monitoring	Clinical research	Increases from cirrhosis to early HCC
Probiotics	Single/multi strain formulations (*Lactobacillus*, *Bifidobacterium*, etc.)	Barrier enhancement; immune modulation; metabolite production	Preclinical & early clinical	Strain-specific effects; host-dependent efficacy; 26 RCTs show reduced inflammation markers
	Prohep (synbiotic formulation)	Promotes *Prevotella*/*Oscillibacter*; inhibits Th17; reduces tumor volume by 40%	Preclinical	Upregulates IL-10, IL-13, IL-27; inhibits VEGF-A
	Synbiotics (GOS, FCT)	Upregulates MUC2, TFF3, tight junction proteins; enriches beneficial bacteria	Preclinical	Increases fecal SCFA levels; downregulates inflammatory markers
Fecal microbiota transplantation (FMT)	From healthy donors	Reshapes microbiome; reduces inflammation; enhances ICI response	Clinical trials ongoing	Effective in alcoholic hepatitis; improves ICI response in non responders; safety concerns (infection risk, lack of standardization)
	From ICI responders to non-responders	Increases antigen presenting cells and tumor-infiltrating lymphocytes	Clinical case studies	Reactivates anti-tumor immunity; enriches *Lachnoclostridium* and *Akkermansia*
Antibiotics	Vancomycin	Selectively reduces secondary bile acid producing microbes (*Clostridium* clusters XI, XIVa)	Preclinical	Inhibits obesity-induced HCC; dual effects: beneficial in suppressing carcinogenic microbes but detrimental when combined with ICI (increased mortality)
	Antibiotic stewardship	Preservation of beneficial microbiota during cancer treatment	Clinical guideline development	Concurrent antibiotic use with ICI associated with poor prognosis (HR ~ 1.46 1.60)
Dietary modulation	High dietary fiber/whole grains	SCFA production; reduces HCC risk by 17% per 10 g/day fiber	Human epidemiology (strong)	22–31% reduction in liver cancer incidence; 56–63% reduction in chronic liver disease mortality
	Prebiotic supplementation (e.g., GOS)	Enhances beneficial bacteria; improves barrier function	Preclinical	Context-dependent: may promote tumorigenesis under dysbiosis
TLR4 antagonists	Polymyxin B; LPS-binding compounds; small molecules; thalidomide derivatives	Inhibit LPS/TLR4/MyD88/NF κB pathway	Preclinical development	Reduces chronic inflammation; potential risk of immune suppression and infection
FXR agonists	Obeticholic acid, GW4064	Maintain intestinal barrier; induce FGF19 release; reduce bacterial translocation	Clinical trials	Improve liver function; prevent barrier dysfunction; reduce inflammation in cirrhosis
Neuro-immune modulation	Targeting microbiota brain-liver axis	GABA/5-HT modulation of HPA axis and immune responses	Conceptual framework	Emerging paradigm; requires clinical validation
Liver-spleen axis intervention	Integrated gut liver-spleen targeting	Address systemic inflammation and splenic immune dysfunction	Preclinical research	Th17/Treg balance; macrophage polarization; novel therapeutic target

AUC, area under the curve; TMAO, trimethylamine N-oxide; RCT, randomized controlled trial; GOS, galacto-oligosaccharides; FCT, fermented milk with *L. gasseri* 505 and *C. tricuspidata*; MUC2, mucin 2; TFF3, trefoil factor 3; VEGF-A, vascular endothelial growth factor A; FMT, fecal microbiota transplantation; ICI, immune checkpoint inhibitor; SCFA, short-chain fatty acids; TLR4, Toll-like receptor 4; LPS, lipopolysaccharide; MyD88, myeloid differentiation primary response 88; FXR, farnesoid X receptor; FGF19, fibroblast growth factor 19; GABA, gamma-aminobutyric acid; 5-HT, serotonin; HPA, hypothalamic–pituitary–adrenal. Clinical Status: Clinical validation—tested in clinical diagnostic or observational studies; Preclinical—under investigation in cell or animal models; Clinical trials ongoing—currently being evaluated in human clinical trials; Clinical research—exploratory clinical studies; Clinical guideline development—evidence supporting clinical practice recommendations; conceptual framework—theoretical foundation requiring clinical validation. Note: HR, hazard ratio. Safety considerations and standardization protocols require further development for clinical implementation. Efficacy may vary based on patient characteristics, disease etiology, and treatment context. Summary: This table synthesizes current evidence on microbiome-targeted therapeutic strategies, ranging from established biomarkers to emerging interventions. Clinical translation requires careful consideration of safety profiles, standardization protocols, and patient-specific factors.

### 3.1 Biomarker and diagnostic tool development

HCC stands as a globally devastating malignancy, with annual mortality surpassing 700,000 cases (Bray et al., 2018). Current diagnostic methodologies predominantly rely on serum alpha-fetoprotein level detection and radiological imaging (Huang et al., 2024). However, the absence of high-accuracy predictive biomarkers, compounded by atypical early-stage symptomatology, results in most patients being diagnosed after the window for curative surgical intervention has closed. Alpha-fetoprotein a conventional predictive marker, suffers from inherent limitations in specificity, demonstrating significant elevations across a spectrum of pathological conditions, including active hepatitis, gonadal germ cell tumors, secondary liver cancer, and pregnancy—severely compromising diagnostic accuracy in HCC (Huang et al., 2024). Consequently, novel diagnostic marker development with heightened specificity holds substantial clinical importance for enhancing early HCC detection rates and improving patient prognosis.

Gut microbiome-based markers have emerged as uniquely advantageous in early HCC diagnosis. A study encompassing 75 patients with HBV infection-associated HCC systematically evaluated microbiome diagnostic utility as a non-invasive biomarker, with findings validated in an independent cohort of 18 patients from diverse geographical regions (Ren et al., 2019). Study data revealed marked fecal microbial diversity increase trend during progression from liver cirrhosis to early-stage HCC. A diagnostic model constructed upon 30 characteristic microbial markers achieved an area under the receiver operating characteristic curve of 80.64% in distinguishing 75 early HCC cases from 105 non-HCC samples (Ren et al., 2019), substantiating gut microbiome marker potential as a non-invasive tool for early liver cancer diagnosis.

### 3.2 Microbiome-mediated modulation of therapeutic efficacy

In-depth investigations into the regulatory mechanisms of the gut-liver axis illuminate novel intervention targets for HCC prevention and treatment. Systematic preclinical research confirms that targeted modulation of the gut-liver axis can markedly reduce HCC incidence in murine models (Li et al., 2024). Current therapeutic strategies targeting the gut microbiome demonstrate considerable clinical translational value. Furthermore, biotherapeutic approaches, such as probiotic formulations and fecal microbiota transplantation (FMT), are gaining traction in clinical practice due to their safety profiles and efficacy (Yu and Schwabe, 2017). Targeted therapeutic strategies focused on the gut-liver axis offer multifaceted clinical benefits. The development of preventive and therapeutic strategies targeting this axis holds substantial clinical translational value for enhancing the quality of life for liver cancer patients.

#### 3.2.1 Fundamental strategies for microbiome modulation

Probiotics are defined as “live microorganisms which, when administered in adequate amounts, confer a health benefit on the host” (Kaźmierczak-Siedlecka et al., 2022). Their mechanisms of action span multiple dimensions. At the level of host physiology and immune regulation, probiotics modulate mucosal immune responses, mediate microbial interactions, and produce metabolic byproducts such as short-chain fatty acids (SCFAs). These effects significantly enhance trans-epithelial resistance (TER), reduce serum levels of zonulin, endotoxins, and lipopolysaccharide (LPS), and downregulate inflammatory markers including C-reactive protein (CRP), tumor necrosis factor-*α* (TNF-α), and interleukin-6 (IL-6). A meta-analysis of 26 randomized controlled trials has confirmed that the synergistic enhancement of barrier function and attenuation of inflammation contributes to intestinal homeostasis and may improve subsequent therapeutic responses (Zheng et al., 2023). At the level of microbial ecology and functionality, probiotics suppress pathogenic bacteria through substrate competition, production of antimicrobial metabolites (e.g., lactic acid, bacteriocins), competitive adhesion, and exclusion mechanisms. These actions, coupled with host-mediated barrier enhancement and immune modulation, promote the enrichment of beneficial taxa such as *Bifidobacterium* and *Lactobacillus*, while inhibiting Gram-negative bacteria-associated inflammatory signaling, thereby optimizing the host’s an ti-inflammatory and immune responses (Zheng et al., 2023; Lee et al., 2020; O’Toole and Cooney, 2008). Moreover, probiotics can biotransform non-nutritive dietary components such as proanthocyanidins into bioactive metabolites with anticancer properties, providing a theoretical basis and research direction for probiotic-based dietary interventions and adjuvant therapies targeting hepatocellular carcinoma (HCC). Evidence from gut–liver axis studies indicates that intestinal barrier disruption and microbial dysbiosis can activate Toll-like receptor 4 (TLR4) signaling via microbe-associated molecular patterns (MAMPs), particularly LPS, thereby driving hepatic inflammation, fibrosis, and oncogenic signaling. In this context, reconstructing a “quasi-healthy” intestinal microbiota and barrier function—such as through probiotic administra tion—is considered a viable strategy for intervening in HCC pathogenesis, with potential benefits demonstrated in animal models and early clinical studies (Yu and Schwabe, 2017). Preclinical evidence further suggests that specific synbiotic/probiotic combinations can directly reinforce the intestinal barrier and at tenuate inflammatory responses. For instance, galacto-oligosaccharides (GOS) have been shown to upregulate MUC2 expression, thicken the colonic mucus layer, and reduce intestinal permeability in murine models, even under antibiotic intervention. These effects are accompanied by partial mod ulation of inflammatory markers (e.g., IL-6, TNF-α gene expression), indicating that GOS may improve barrier homeostasis by reshaping microbial functionality and host epithelial transcription (Kuo et al., 2021). The synbiotic FCT (fermented milk with *L. gasseri* 505 and *C. tricuspidata*) has demonstrated concurrent upregulation of MUC2, TFF3, and tight junction proteins (occludin, ZO-1), along with significant downregulation of TNF-α, IFN-*γ*, IL-1β, IL-6, and iNOS/COX-2 in colitis-associated cancer models. These changes are accompanied by enrichment of *Lactobacillus*, *Bifidobacterium*, and *Akkermansia*, as well as elevated fecal SCFA levels (acetate, propionate, butyrate), indicating coordinated improvement along the “microbiota–barrier–inflammation” axis (Roehlen et al., 2023). In avian models, *Lactobacillus plantarum* GX17 significantly upregulated CLDN, MUC2, and TLR2 expression and optimized villus/crypt morphology, supporting a conserved mechanism for enhancing epithelial tight junctions and mucosal innate recognition, consistent with mammalian data (Arnold et al., 2021). Importantly, SCFAs act as epigenetic mediators between the microbiota and host, inhibiting histone deacetylases (HDACs), increasing H3/H4 acetylation, and promoting IL-22 and regulatory T cell (Treg) path ways, thereby shaping a systemic anti-inflammatory immune phenotype. At both intestinal and hepatic levels, SCFAs remodel chromatin states and transcriptional rhythms, suggesting that a “SCFA-enriched microbiota” may epigenetically suppress TLR/LPS-driven proinflammatory signaling, improve the inflammatory microenvironment of the gut–liver axis, and enhance HCC treatment sensitivity (Woo and Alenghat, 2022).

Despite the considerable potential demonstrated in mechanistic studies, the clinical efficacy of probiotics exhibits significant variability, primarily stemming from the complex interplay of host-specific characteristics, strain-functional specificity, and disease states combined with treatment contexts. Firstly, host factors including age, baseline gut microbiota composition, immune status, and dietary structure significantly influence probiotic colonization capacity and functional expression. For instance, infants versus adults or healthy individuals versus cancer patients may show markedly different responses to the same probiotic intervention, indicating that efficacy is highly dependent on the host’s inherent gut ecological niche and immune microenvironment (Lu et al., 2021). Secondly, probiotic actions demonstrate clear strain specificity; even different strains within the same species may exhibit substantial functional differences in immune modulation, apoptosis induction, or short-chain fatty acid production. Research shows that *Lactobacillus rhamnosus*GG and *Lactobacillus casei* Shirota operate through distinct mechanisms in regulating cytokine profiles and inhibiting tumor signaling pathways, further emphasizing a “strain-dependent” rather than “species-general” effect (Naz and Zafar, 2025). Additionally, the baseline gut microbial diversity of the host is a critical factor influencing probiotic colonization and functional performance. Individuals with higher microbiome diversity are more receptive to exogenous probiotics and exhibit synergistic effects, whereas dysbiotic states (e.g., in cancer patients after antibiotics or chemotherapy) may trigger “colonization resistance,” significantly attenuating probiotic bioactivity (Ahmad et al., 2025). Finally, cancer type, disease stage, and combination therapies (e.g., chemotherapy or immune checkpoint inhibitors) also modulate the immunoregulatory effects of probiotics. For example, under immunosuppressive conditions, certain probiotics may enhance anti-tumor immune responses, while showing limited effects in immunocompetent individuals; moreover, synergistic effects between probiotics and chemo−/immunotherapies may fluctuate with dynamic changes in the tumor microenvironment (Lu et al., 2021; Ahmad et al., 2025). In summary, the clinical effects of probiotics are not constant but result from multifactorial interactions. Future application strategies should adhere to individualized principles, employing precision matching based on host microbial profiles, disease status, and strain-specific functions to enhance intervention efficacy and safety.

#### 3.2.2 Micro-ecological system reconstruction

FMT as a novel strategy for micro-ecological system reconstruction, is increasingly demonstrating its value in HCC therapy. Systematic research confirms that FMT mitigates hepatic inflammatory responses by reshaping the compositional structure of the gut microbiome, thereby reducing the risk of HCC development (Bajaj et al., 2022). This microbial restructuring directly modulates the meta-metabolome—a dynamic pool of microbial metabolites that serve as functional biomarkers for precision dietary interventions, thereby bridging microbial composition shifts to host metabolic phenotypes (Luo and Wang, 2025). In a high-fat diet-induced non-alcoholic steatohepatitis model, FMT not only significantly inhibited intrahepatic lipid accumulation but also reduced the expression levels of pro-inflammatory cytokines, such as interferon-*γ* and IL-17 (Zhou et al., 2017). Clinical studies indicate that, for alcoholic hepatitis patients refractory to corticosteroids, FMT from healthy donors can effectively ameliorate severe liver disease manifestations, including ascites and hepatic encephalopathy, while concurrently reducing inflammatory marker levels (Philips et al., 2017).

The synergistic role of FMT in immune checkpoint inhibitor (ICI) therapy has been extensively validated. Clinical observations reveal a marked increase in mortality rate among patients receiving combined antibiotic and ICI treatment, underscoring the pivotal role of the gut microbiota in modulating ICI therapeutic outcomes (Pinato et al., 2023; Cheung et al., 2023). FMT enhances the levels of treatment response-associated bacterial strains and metabolites by reconstituting gut microbiome composition. Clinical research data demonstrate that fecal transplantation from programmed cell death protein 1 blockade therapy responders into non-responders can significantly enhance the latter’s therapeutic responsiveness to programmed cell death protein 1 blockade (Davar et al., 2021). This therapeutic effect is closely associated with increased infiltration of antigen-presenting cells and tumor-infiltrating lymphocytes in the gut, confirming the reactivation of anti-tumor immune responses (Baruch et al., 2021). While FMT research in HCC therapy remains exploratory, existing experimental evidence corroborates its enrichment effect on beneficial flora, potentiating ICI efficacy. Studies find that selective enrichment of *Lachnoclostridium* is significantly correlated with increased secondary bile acid levels—ursodeoxycholic acid, tauro-ursodeoxycholic acid, uric acid, and murideoxycholic acid—promoting HCC responsiveness to ICI therapy (Lee et al., 2022). Quantitative targeted meta-metabolomics, leveraging platforms such as LC–MS/MS with stable isotope labelling, can delineate flux dynamics of key metabolites (e.g., SCFAs and bile acids), thus uncovering mechanistic pathways underlying FMT efficacy (Luo and Wang, 2025). Furthermore, *Akkermansia muciniphila* colonization exhibits a strong correlation with ICI treatment sensitivity across a range of solid malignancies, including HCC (Zheng et al., 2019).

However, the clinical application of FMT continues to face significant safety challenges. Inadequate donor screening protocols can lead to pathogen transmission, with documented cases of fatal infections caused by multidrug-resistant bacteria (e.g., ESBL-producing *E. coli*) due to insufficient screening, posing an extremely high risk to immunocompromised HCC patients (Drew, 2024; Xu et al., 2022). Furthermore, the long-term consequences of FMT remain unclear, carrying potential risks of inducing unknown immune abnormalities or metabolic disturbances, and there is currently a complete lack of standardized preparations and administration protocols, resulting in poor reproducibility of efficacy (Drew, 2024; Wardill et al., 2019). Retrospective and clinical studies indicate that transient gastrointestinal reactions, such as diarrhea and abdominal distension, are common after FMT, while more severe adverse events include aspiration pneumonia and gastrointestinal bleeding associated with enema or endoscopic procedures (Wardill et al., 2019). Consequently, FMT must be conducted within a stringent regulatory framework, and there is an urgent need for well-designed, large-scale clinical trials specifically in HCC populations to comprehensively evaluate its risk–benefit ratio (Drew, 2024; Xu et al., 2022; Wardill et al., 2019).

#### 3.2.3 Antibiotics

Antibiotics exhibit complex bidirectional regulatory effects in HCC development by modulating gut microbiota composition and metabolite networks (Ma et al., 2018; Singh et al., 2020). On one hand, antibiotics can attenuate HCC progression by inhibiting pathogenic microorganism proliferation and reducing intestinal inflammatory responses. Conversely, overuse or abuse of antibiotics may precipitate significant reductions in beneficial gut flora, leading to microecological dysbiosis, thereby potentiating chronic hepatitis and liver cancer progression (Rajapakse et al., 2023). Studies indicate that antibiotic interventions can influence hepatocarcinogenesis and progression via diverse mechanisms. In obesity-induced HCC models, oral antibiotic cocktails can selectively reduce the abundance of symbiotic gut bacterial communities, particularly of certain secondary bile acid-producing microbes, such as *Clostridium* clusters *XI* and *XIVa*, thereby inhibiting DNA damage mediated by these metabolites and diminishing chronic hepatic inflammatory responses (Singh et al., 2020). Mechanistically, secondary bile acids, including deoxycholic acid promote liver fibrosis and tumor progression by augmenting hepatic stellate cell activation and senescence, whereas antibiotic interventions can substantially mitigate these adverse effects (Ma et al., 2018; Singh et al., 2020; Yoshimoto et al., 2013).

However, the role of antibiotics in immunotherapy, particularly in the context of ICI therapy, exhibits negative implications. A systematic review encompassing 9 multi-center clinical trials and nearly 4,100 HCC patients indicated that antibiotic exposure was significantly associated with poor prognosis, regardless of whether patients received ICI, tyrosine kinase inhibitors, or placebo (Pinato et al., 2023). Another prospective study of 395 HCC patients undergoing ICI therapy further corroborated that concurrent antibiotic use during immunotherapy correlated significantly and positively with mortality risk in advanced HCC patients (Cheung et al., 2023). This phenomenon likely arises from antibiotics clearing beneficial gut flora, concurrently attenuating gut microbiota-mediated immune system modulation. Particularly during ICI utilization, gut flora imbalance can impair T cell function and gut-associated immune responses, thereby diminishing patient immunotherapy responsiveness. Judicious antibiotic use may aid in decelerating liver cancer progression, especially in scenarios involving the suppression of harmful microorganisms and the reduction of inflammatory responses. However, antibiotic abuse, particularly prolonged use, may disrupt gut microecological balance, leading to immune dysregulation, thereby accelerating hepatocarcinogenesis and immunotherapy resistance. Consequently, clinical application of antibiotics necessitates heightened precision and individualization.

Antibiotic abuse (particularly when combined with immune checkpoint inhibitors) poses significant risks in cancer treatment, primarily manifested through reduced efficacy of immunotherapy and increased incidence of immune-related adverse events (irAEs). According to multiple clinical studies and meta-analyses, antibiotic exposure is significantly associated with shortened progression-free survival (PFS) and overall survival (OS) in cancer patients receiving anti-PD-1/PD-L1 therapy, with a PFS hazard ratio (HR) of 1.60 (95% CI: 1.33–1.92, *p* < 0.00001) and an OS HR of 1.46 (95% CI: 1.32–1.61, *p* < 0.00001). Similar negative correlations have been observed across various cancer types, including non-small cell lung cancer (NSCLC), renal cell carcinoma (RCC), and melanoma (Jiang et al., 2022). Concurrently, antibiotic use significantly elevates the risk of irAEs, with a pan-cancer analysis indicating an increased irAE risk (OR = 2.12, 95% CI: 1.38–3.22), particularly among lung cancer patients (OR = 3.16, 95% CI: 1.67–5.95). This risk has been validated across multiple pharmacovigilance databases, including FAERS (OR = 1.39, 95% CI: 1.21–1.59) and VigiBase (OR = 1.32, 95% CI: 1.09–1.59) (Jing et al., 2022). Mechanistically, antibiotics disrupt gut microbial diversity (e.g., by reducing the Inverse Simpson index), diminish beneficial bacterial populations (such as *Bifidobacterium* and *Lactobacillus*), and inhibit the production of immunomodulatory metabolites like short-chain fatty acids, thereby impairing antitumor immune responses and promoting the release of inflammatory cytokines (e.g., IL-6, TNF-*α*). These effects lead to T-cell overactivation and tissue damage (Jiang et al., 2022; Jing et al., 2022). Organ-specific analyses further demonstrate that antibiotic abuse is significantly associated with irAEs such as pneumonia (OR = 1.71), thyroid dysfunction (OR = 2.18), hematologic toxicities (e.g., thrombocytopenia and neutropenia), and hepatic or renal dysfunction (Jing et al., 2022). Clinical implications emphasize the necessity of rigorously evaluating antibiotic use during immunotherapy, prioritizing narrow-spectrum antibiotics or alternative anti-infection strategies to preserve gut microbiota function and reduce irAE risk. For patients requiring antibiotics, simultaneous monitoring of microbial composition and immune indicators is recommended, along with exploring protective interventions such as probiotics or microbiota transplantation (Jiang et al., 2022; Jing et al., 2022).

#### 3.2.4 TLR4 antagonists

The TLR4 signaling pathway plays a critical regulatory role in liver cancer progression and represents a core molecular mechanism in the pathogenic effects of the gut-liver axis (Schneider et al., 2022). Given the TLR4 signaling pathway’s pivotal role in hepatopathy pathogenesis and progression, the development of pathway-specific antagonists has become a key research direction for HCC prevention and treatment. Existing TLR4 antagonists can be categorized into four classes based on their mechanisms of action: (1) compounds that bind and sequester LPS, such as polymyxin B; (2) compounds that inhibit interactions between LPS-binding protein and the CD14-LPS complex (Piazza et al., 2009); (3) specific inhibitors targeting the LPS-MD-2 or LPS-MD-2-TLR4 complex; and (4) small molecule compounds that directly target TLR4. Furthermore, studies have identified thalidomide compounds as possessing TLR4-inhibiting activity (Peri and Piazza, 2012). Although TLR4 antagonists show considerable promise for liver cancer therapy, clinical application still faces challenges. First, TLR4 is a crucial component of the host immune system; excessive TLR4 inhibition may lead to broad immune system suppression and an increased risk of infection. Second, TLR4 roles may vary across different immune cell types; therefore, further in-depth research is needed to determine how to specifically target TLR4 signaling in the liver without disrupting systemic immune function.

### 3.3 Maintenance of gut-liver axis function

As a key functional structure of the gut-liver axis, the intestinal barrier exhibits a close relationship with hepatocarcinogenesis. Targeted strategies for intestinal barrier modulation have become a research hotspot, capable of forming a systemic treatment network in conjunction with microbiome modulation and liver-targeted therapies. Bile acids, as core regulatory molecules of the intestinal barrier, play a pivotal role in maintaining intestinal barrier function and homeostasis. The farnesoid X receptor (FXR), a specific receptor for bile acids, not only mediates bile acid regulation of the intestinal epithelial barrier but also participates in crucial physiological processes, including bile acid synthesis inhibition, modulation of hepatic inflammatory responses, promotion of liver regeneration, and suppression of tumor growth (Schaap et al., 2014). Investigations into molecular mechanisms reveal that hepatic effects of FXR are primarily achieved by activating intestinal FXR receptors and inducing the release of fibroblast growth factor 19 (Thomas et al., 2008; Degirolamo et al., 2015). FXR gene knockout mice exhibit impaired intestinal integrity, with pathological changes further exacerbated after bile duct ligation, alongside a higher incidence of HCC (Degirolamo et al., 2015). FXR-specific agonists GW4064 and obeticholic acid can markedly ameliorate mucosal damage, increased ileal barrier permeability, bacterial overgrowth, and bacterial translocation in rodent models (Verbeke et al., 2015; Úbeda et al., 2016), potentially serving as therapeutic agents for HCC prevention.

## 4 Conclusion

The gut microbiome and its metabolites play a critical role in the pathogenesis and progression of hepatocellular carcinoma (HCC) through a multitude of molecular mechanisms, demonstrating a defining dual regulatory function. Enrichment of specific microbial communities, such as *Proteobacteria*and *Actinobacteria*, along with metabolites like trimethylamine N-oxide (TMAO) and secondary bile acids, promotes hepatocarcinogenesis by modulating the tumor microenvironment, activating oncogenic signaling pathways, and inducing epigenetic alterations. Conversely, beneficial bacteria, including *Lactobacillaceae*and *Bifidobacterium*, and metabolites such as short-chain fatty acids (SCFAs), confer protection by enhancing intestinal barrier integrity, regulating immune homeostasis, and suppressing key oncogenic pathways. These insights significantly advance our understanding of the gut-liver axis in HCC and provide a robust foundation for developing microbiome-based diagnostic biomarkers and targeted therapeutics.

Despite these advances, several challenges impede clinical translation. The causal relationship between specific microbial strains and HCC pathogenesis requires further elucidation. The functional networks and overarching regulatory mechanisms of microbial metabolites need systematic dissection. Furthermore, the diagnostic validity of microbial biomarkers lacks support from large-scale, multicenter clinical studies, and the development of personalized treatment regimens is constrained by a scarcity of high-level clinical evidence.

Confronting these challenges should be the priority for future research. Key directions include: (1) the integration of multi-omics data with advanced artificial intelligence algorithms to decipher complex host–microbe interactions and identify high-fidelity biomarker panels for early detection and prognostic prediction; (2) the execution of meticulously designed longitudinal cohort studies and interventional trials to definitively establish causality and validate the efficacy of microbiome-targeting interventions; and (3) the development of precision strategies targeting specific microbial strains, encompassing optimized fecal microbiota transplantation (FMT) protocols and engineered next-generation probiotics. A concerted effort along these avenues is poised to usher in a new era of microbiome-guided precision medicine for the prevention and management of HCC.
